# *Rhodococcus* as Biofactories for Microbial Oil Production

**DOI:** 10.3390/molecules26164871

**Published:** 2021-08-11

**Authors:** Héctor M. Alvarez, Martín A. Hernández, Mariana P. Lanfranconi, Roxana A. Silva, María S. Villalba

**Affiliations:** INBIOP (Instituto de Biociencias de la Patagonia), Consejo Nacional de Investigaciones Científicas y Técnicas (CONICET), Facultad de Ciencias Naturales y Ciencias de la Salud, Universidad Nacional de la Patagonia San Juan Bosco, Ruta Provincial N° 1, Km 4-Ciudad Universitaria, Comodoro Rivadavia 9000, Chubut, Argentina; mahernandez@unpata.edu.ar (M.A.H.); mlanfranconi@unpata.edu.ar (M.P.L.); rsilva@unpata.edu.ar (R.A.S.); msol_villalba@yahoo.com (M.S.V.)

**Keywords:** *Rhodococcus*, single-cell oil, biofactory

## Abstract

Bacteria belonging to the *Rhodococcus* genus are frequent components of microbial communities in diverse natural environments. Some rhodococcal species exhibit the outstanding ability to produce significant amounts of triacylglycerols (TAG) (>20% of cellular dry weight) in the presence of an excess of the carbon source and limitation of the nitrogen source. For this reason, they can be considered as oleaginous microorganisms. As occurs as well in eukaryotic single-cell oil (SCO) producers, these bacteria possess specific physiological properties and molecular mechanisms that differentiate them from other microorganisms unable to synthesize TAG. In this review, we summarized several of the well-characterized molecular mechanisms that enable oleaginous rhodococci to produce significant amounts of SCO. Furthermore, we highlighted the ability of these microorganisms to degrade a wide range of carbon sources coupled to lipogenesis. The qualitative and quantitative oil production by rhodococci from diverse industrial wastes has also been included. Finally, we summarized the genetic and metabolic approaches applied to oleaginous rhodococci to improve SCO production. This review provides a comprehensive and integrating vision on the potential of oleaginous rhodococci to be considered as microbial biofactories for microbial oil production.

## 1. Introduction

Some eukaryotic microorganisms, such as fungi, microalgae, and yeasts, can be considered oleaginous species because they are able to produce more than 20% (of cellular dry weight, CDW) of triacylglycerols (TAG) [1]. They exhibit some differential metabolic features compared to non-oleaginous related species, such as a high flux of acetyl-CoA and NADPH to fatty acid and TAG biosynthetic pathways during the cultivation of cells under nitrogen-limiting conditions in the presence of an excess of the carbon source [2]. These oleaginous microorganisms have been considered as alternative sources of single-cell oils (SCO) with potential applications in the industry. According to the fatty acid composition of the produced oils, they may serve as ingredients or components in many manufactured products, such as biofuels, biolubricants, soaps, cosmetic, nutritional or pharmaceutical products, paints, or oleochemicals, among others [3]. The application of microbial oils in the industry is a promising avenue, although the costs of lipid production at a large industrial scale are currently very high. For this reason, the main problem to be solved is the reduction of SCO production costs. The combination of fundamental knowledge on the biology of oil-producing microorganisms, with innovations in fermentation and strain engineering processes, will contribute to the economic feasibility of microbial oil production at a large industrial scale.

The accumulation of significant amounts of SCO is not restricted to eukaryotic microorganisms as prokaryotes are also able to produce TAG as storage lipids. Previous studies revealed that Gram-negative as well as Gram-positive bacteria are able to produce variable amounts of TAG during the cultivation of cells on different carbon sources [3]. In general, TAG represent only minor components of the stored lipids in Gram-negative bacteria, whereas wax esters (WE), esters of long-chain fatty alcohols with long-chain fatty acids, are the main lipid compounds accumulated by these microorganisms [3]. Among prokaryotes, Gram-positive actinobacteria seem to be specialized in the accumulation of TAG as main storage lipids [4,5,6]. Some species are able to accumulate very high levels of TAG in their cells (more than 50% CDW), such as some strains belonging to *Streptomyces* or *Rhodococcus* genera [7,8,9]. *Rhodococcus opacus* PD630 and *R. jostii* RHA1 have been the preferred research models among oleaginous actinobacteria. Different study strategies have been applied to elucidate the biology of these oleaginous bacteria, such as “omic” approaches (genomic, transcriptomic, proteomic, and metabolomic analyses) or the characterization of specific genes/proteins involved in lipogenesis, such as (i) enzymes of central and lipid metabolisms; (ii) enzymes for the catabolism of various substrates; (iii) lipid transporter proteins; (iv) proteins associated with lipid droplets; and (v) proteins involved in the regulation of gene expression. In addition, several studies have been focused on the ability of oleaginous rhodococci to produce SCO from industrial or municipal wastes with a perspective oriented to the biotechnological application of the process. Finally, different genetic approaches have been applied to oleaginous rhodococci to (i) increase the yield of SCO production, (ii) expand the range of application of these bacteria to use new substrates for the production of oils, and (iii) produce novel value-added lipids.

This expanding body of knowledge demonstrates that oleaginous rhodococci exhibit several intrinsic properties that make them efficient biofactories for SCO production. This review provides a conceptual framework connecting the results of different studies for the elaboration of an unified and integrated view on the potential of oleaginous rhodococci as platforms for the production of SCO at industrial scale.

## 2. Biological Properties of Oleaginous *Rhodococci*

**Figure 1:**
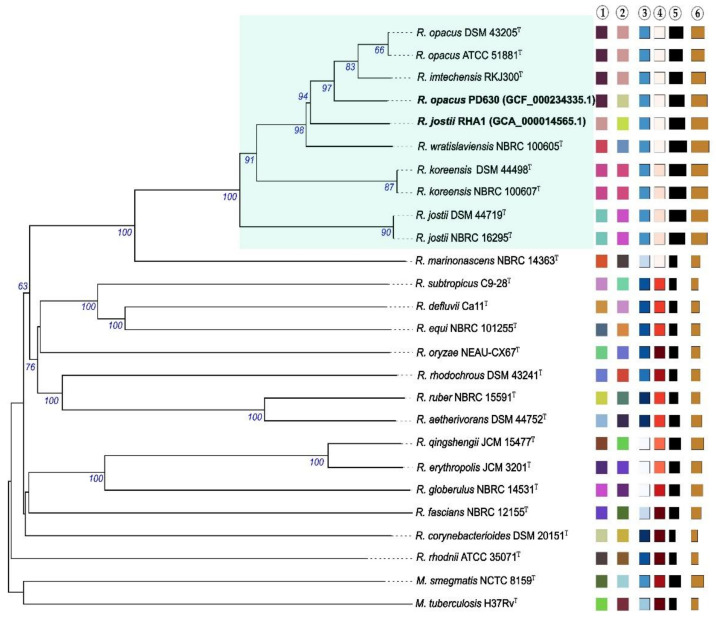
Phylogenomic tree inferred using the Type Strain Genome Server (https://tygs.dsmz.de/ accessed on 13 February 2021) by Genome BLAST Distance Phylogeny approach (GBDP), using distances calculated from genome sequences. The branch lengths are scaled in terms of GBDP distance formula *d5*. The numbers below branches are GBDP pseudo-bootstrap support values >60% from 100 replications, with an average branch support of 79.8%. The tree was rooted at the midpoint. The light blue box highlights *Rhodococcus* species (including oleaginous strains RHA1 and PD630) belonging to the group C, according to the classification proposed by Sangal et al. [10]. Leaf labels are annotated by affiliation to ① species and ② subspecies clusters, predicted by the digital DNA:DNA hybridization (dDDH) calculator service; ③ genomic G+C content (Min. 61.7% 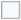, Max. 70.7% 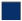); ④ δ values (Min. 0.1 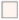, Max. 0.3 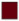); ⑤ overall genome sequence length (Min. 3.9 Mb 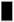, Max. 10.4 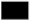); and ⑥ number of proteins (Min. 3611 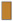, Max. 9472 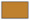).

Phylogenetic and taxogenomic studies revealed that members of the genus *Rhodococcus* make up a heterogenous taxon, including at least seven rhodococcal species-groups [10]. Interestingly, there is a high genetic and metabolic heterogeneity between rhodococcal species, which makes the potential application of these microorganisms in different industrial processes very attractive. Although the ability to synthesize TAG is widely distributed among the different species of the genus, the accumulation of significant amounts of TAG (oleaginous rhodococci) seems to be restricted to the species-group C (*R. opacus* cluster), according to the group classification proposed by Sangal et al. [10]. This species-group includes environmental isolates belonging to *R. opacus*, *R. jostii*, *R. wratislaviensis*, and *R. imtechensis* (Figure 1).

These bacteria possess large genomes (between 7.8–10.4 Mb) and a wide metabolic repertoire that seems to have specialized throughout evolution to convert different substrates into lipids that are intracellularly accumulated in large quantities. The metabolic machinery of these microorganisms includes a set of enzymes and pathways promoting the efficient production of energy, reducing the equivalents (NADPH) and metabolic precursors (acetyl-CoA, acyl-CoAs, glycerol-3-phosphate, among others) needed for the biosynthesis and accumulation of TAG in the form of lipid droplets. Under nitrogen-limiting conditions, which stimulate TAG synthesis and accumulation, oleaginous rhodococci significantly up-regulate Entner–Doudoroff (ED) and pentose–phosphate (PP) pathways [11,12]. These routes provide reducing equivalents (NADPH) required as a cofactor for fatty acid biosynthesis and metabolic precursors for lipogenesis. Additionally, Group C species possess multiple NADP^+^-dependent enzymes, reactions, and pathways, which are activated to provide the necessary cofactor pools for massive lipid biosynthesis, such as non-phosphorylating glyceraldehyde-3-phosphate dehydrogenase (GAPN), NADP^+^-malic enzymes, and NADP^+^-dependent succinate-semialdehyde dehydrogenase, among others [13,14,15]. The available evidence suggests that oleaginous rhodococci possess a potent NADPH-producing system that supports the high demand of cofactors for massive lipogenesis under nitrogen-limiting conditions. Oleaginous bacteria usually promote profound changes in the metabolism with a strong redirection of the carbon flux to lipid biosynthesis. The fluxes of carbon metabolism must be adjusted and optimized during intensive lipogenesis probably by a multi-level regulation system. The metabolic reorganization in oleaginous rhodococci seems to include the down-regulation of reactions and pathways that consume NADPH and precursors needed for lipid biosynthesis, such as amino acid and nucleotides synthesis, L-ectoine production, or glycogen biosynthesis, among others. Major changes in the central carbon metabolism also occur during TAG accumulation in oleaginous rhodococci. Transcriptomic and proteomic analyses [11,12,16] suggested that the metabolic strategy with full respiration to gain energy during cell growth must be changed to another metabolic scenario by reducing the tricarboxylic acid cycle (TCA cycle) and activating the glyoxylate pathway when nitrogen availability decreases. Thus, the sink for TCA cycle metabolites is depleted in cells, and acetyl-CoA, the source of carbon for the cycle, can be shunted toward fatty acid biosynthesis. In addition, the reduction of the TCA cycle and concomitantly the cellular respiratory activity in cells reduces the generation of reactive oxygen species (ROS) by respiration, reducing the demand for available NADPH pools by the antioxidant defense systems to sustain lipogenesis [15]. On the other hand, oleaginous rhodococci such as *R. jostii* RHA1 possess another mechanism based on a much faster enzyme level regulation to reroute carbon fluxes to sustain TAG accumulation during nitrogen deprivation. These bacteria possess a glycogen-recycling system that allows modulating the carbon fluxes into glycolytic pathways according to their metabolic cell demands [17]. Glycogen biosynthesis in *R. jostii* RHA1 is allosterically regulated through the activity of the ADP-glucose pyrophosphorylase (GlgC) enzyme depending on the availability of different effector molecules of sugar and amino sugar metabolisms [17,18]. The combined availability of these effectors (activators and inhibitors of GlgC activity) orchestrates the tight regulation of the enzyme and glycogen biosynthesis in this microorganism. Interestingly, 6 P-gluconate (one of the key ED pathway products) and NADPH (required for fatty acid synthesis) down-regulate glycogen synthesis in RHA1, increasing the intracellular availability of carbon for lipogenesis. Recently, Cereijo et al. postulated a new metabolic node in *R. jostii* RHA1 involved in the partitioning of glucosamine-1P, which was a substrate for at least three enzymes (GalU2, GlmU, and GlgC), in the cell metabolism [19]. This node may be considered a metabolic innovation in oleaginous rhodococci for connecting carbon and nitrogen metabolisms and coupling physiological functions such as cell-wall synthesis, cell growth, or TAG accumulation, according to the environmental conditions.

The accumulation of TAG under nitrogen deprivation in rhodococci is not only a consequence of the up-regulation of TAG biosynthesis genes but also of the reallocation of carbon and nitrogen resources in the cellular metabolism. Carbon and nitrogen metabolisms are closely connected in bacterial cells; thus, carbon and nitrogen reallocation in response to nitrogen deprivation might play a key role in providing precursors for TAG synthesis in rhodococci. In this context, a transcriptional regulatory protein (called NlpR) that simultaneously modulates carbon and nitrogen metabolisms has been reported for the oleaginous *R. jostii* RHA1 and *R. opacus* PD630 in response to nitrogen limitation [20]. The NlpR regulator in oleaginous rhodococci provides a stronger redirection of carbon fluxes toward lipid metabolism, improving lipogenesis specifically under nitrogen limitation. This regulatory protein modulates the allocation of carbon into different lipid fractions, such as TAG, DAG, MAG, fatty acids, phospholipids, and glycolipids in response to nitrogen levels [21]. Interestingly, recent studies confirmed that the regulon of the orthologous transcriptional regulator (called NnaR) in *Mycobacterium smegmatis* is only restricted to genes involved in nitrogen metabolism but not in lipogenesis [22]. The amplification of NlpR regulon in oleaginous rhodococci in comparison to other non-oleaginous actinobacteria might help cells to modulate carbon and nitrogen reallocation more finely according to the level of available nitrogen source and to enhance the ability to accumulate TAG under nitrogen-limiting conditions. NlpR could be part of a complex regulatory network shaped during the evolution of these microorganisms to interconnect the regulation of carbon and nitrogen metabolisms.

It is clear that among rhodococci, at least one species-group evolved as natural cell factories for hyper-lipid production. Oleagenicity in these microorganisms seems to be supported by complex and orchestrated interactions among transcriptional, translational, posttranslational, and metabolic mechanisms. This conservative metabolic strategy might represent an advantage for surviving in nutrient-poor environments, such as deserts or arid ecosystems where these microorganisms are frequently found.

## 3. Comparison of Rhodococci to Other Oleaginous Microorganisms

Microbial lipids, also known as single-cell oils (SCO), are highly attractive feedstocks for the production of diverse value-added products, such as biofuels, additives for feed and cosmetics, oleochemicals, lubricants, and other manufactured products. In addition, oleaginous microorganisms are interesting sources for SCO production due to their fast production rates, independence from seasonal and climatic changes, and ease of scale-up for industrial processing. Although several oleaginous microorganisms have been identified and explored for their ability to produce TAG, including microalgae, yeasts, fungi, and bacteria, each species exhibits intrinsic features and specific physiological/biochemical repertoires to produce high oil levels. In this context, it is interesting to do a comparative overview of rhodococci in relation to other oleaginous microorganisms (Table 1).

In general, the physiological capacity of microorganisms to produce SCO is variable and depends mainly on intrinsic properties linked to their genetic and enzymatic machinery and regulatory mechanisms and on extrinsic properties such as the conditions used for cell cultivation. Moreover, microorganisms can be differentially amenable to genetic and metabolic engineering procedures to improve the oil production process.

Some rhodococcal species are natural-oil producers with the ability to accumulate very high amounts of TAG ranging between 50% to 75% (of CDW) from single-carbon sources, such as gluconate, glucose, or benzoate [8,16]. In addition, some strains of *R. opacus*, *R*. *wratislaviensis*, or *R. jostii* exhibited a TAG content of approximately 77–87% (CDW) after the cultivation of cells on olive oil or olive mill wastes as carbon sources [8,43]. Only a few oil-natural microbial producers have shown this extraordinary ability to accumulate lipids at high levels, usually after culturing on mixed substrates (e.g., glucose/xellobiose/xylose/glycerol) or waste-based culture media (Table 1) [44,45].

Juanssilfero et al. reported the ability of *Lipomyces starkeyi*, an oleaginous yeast, to produce up to 84.6% (*w*/*w*) from a mixture of glucose/xylose after high-density inoculation [30]. In another study, Papanikolaou et al. described the accumulation of high amounts of lipids (83.3%, *w*/*w*) by the fungus *Mortierella isabellina* ATHUM 2935 after 4 days of cell cultivation on glucose [39]. On the other hand, some microalgae have been reported as oil-producers at high levels, including *Botryococcus braunii* (75%, *w*/*w*), *Neochloris oleoabundans* (54%, *w*/*w*), and *Schizochytrium* sp. (77%, *w*/*w*), after cell cultivation under variable growth conditions (Table 1) [46,47]. Genetic manipulation of the oleaginous microalga *Neochloris oleoabundans* resulted in an increase in total lipid content of up to 79% (*w*/*w*) [48].

Other features to be considered for technological approaches using oil-producing microorganisms are the cellular biomass yields and growth rates. In general, oleaginous rhodococci exhibit fast growth and high levels of cellular biomass production depending on the carbon source used for cell cultivation (Table 1). Rhodococcal cells usually reach a stationary growth phase, where lipid accumulation is maximal, after hours or days (48–72 h) of cultivation on diverse carbon sources, such as glucose, gluconate, or whey. Eukaryotic microorganisms exhibit variable growth rates and cellular biomass yields depending on the conditions used for cell cultivation (e.g., autotrophic or heterotrophic conditions), but in general they need longer time periods to reach their maximal lipid contents (Table 1). Archanaa et al. performed an interesting study comparing the oleaginous capacities of *R. opacus* PD630 and *Chlorella vulgaris* NIOT5 and their properties as sustainable biodiesel feedstocks [49]. They reported that the bacterial growth rate, biomass productivity, lipid accumulation, and biodiesel productivity were 25-fold, 57-fold, 14-fold, and 75-fold higher, respectively, in comparison to the microalgal strain. Furthermore, according to the chemical properties and fatty acid composition of the extracted oils, the biodiesel obtained from *R. opacus* oils exhibited higher quality compared to that from *Chlorella vulgaris* (Table 1) [49].

The composition of fatty acyl residues in the SCO is another differential property when comparing rhodococcal and eukaryotic oil-producing systems, although the fatty acid composition of microbial lipids depends on the substrate used as a carbon source for cell cultivation, to some extent. In general, oleaginous rhodococci mainly produce saturated and monounsaturated, even- and odd-numbered fatty acids with a chain-length between C_14_ to C_18_ (Table 1). This fatty acid profile makes rhodococcal oils good feedstocks for biodiesel production. Unlike eukaryotic oil-producers, rhodococci are not able to produce TAG containing long chain-length or polyunsaturated (PUFA) fatty acids, which can be used mainly for nutritional and pharmaceutical applications. Long polyunsaturated fatty acids, i.e., docosahexaenoic acid (22:6, ω-3) and arachidonic acid (20:4, ω-6), are produced by oleaginous fungi such as *Mortierella alpine* or *Mucor circinelloides* and different oleaginous microalgae (Table 1) [1]. However, different engineering strategies have been suggested for rhodococci to modify the composition of the accumulated TAG to expand their field of application. Blakie et al. proposed *R. opacus* PD630 as a host strain of polyunsaturated fatty acid synthase genes (*pfa* genes from marine bacterial strain of *Shewanellabaltica*) expression for PUFA production [50].

The yield and composition of microbial SCO depend on the type of fermentation and the particular conditions (e.g., medium, pH-value, temperature, aeration, nitrogen source). Autotrophic microalgae have been considered as promising substitutes for oil seed crops (it has been claimed to be up to 20 times more productive than oil seed crops); however, scaling up and the low biomass and oil productivities are still the main obstacles to overcome [51]. On the other hand, although yeasts usually exhibit higher growth rates than microalgae [2,52], industrial-scale cultivation is often associated with bacterial contamination, resulting in yeast growth inhibition and low yield and lipid productivity [53]. In this context, rhodococci exhibit, in general, high resistance to several stress conditions, such as metal toxicity, UV radiation, high concentration of organic solvents, low temperatures, high salinities, and alkalinity, among others, which may expand their application to different biotechnological platforms purposes even under changing growing conditions [54]. The possibility of cultivating cells under more restricted conditions in a biotechnological process, which can reduce the replication of other contaminating microorganisms, could be one of the advantages of using rhodococci as sources of microbial oils.

A common feature of oleaginous microorganisms, including bacteria, microalgae, fungi, and yeasts, is their ability to generate a continuous supply of acetyl-CoA and NADPH for the fatty acid production under nutrient-limited (usually nitrogen limitation) but carbon-excess conditions. The metabolic and molecular mechanisms involved are intrinsic properties of each organism. The main pathway for TAG biosynthesis in oleaginous microorganisms occurs through three sequential acyl transfers from acyl-CoA to a glycerol backbone (Kennedy pathway). In the final step of TAG biosynthesis (third acylation reaction), an acyl-residue is transferred to the vacant position of diacylglycerol. This reaction, which is the only dedicated step in TAG synthesis, is catalyzed by a diacylglycerol acyltransferase enzyme (DGAT). Interestingly, prokaryotic DGAT enzymes (including rhodococcal enzymes) are a new class of TAG-producing enzymes, which exhibit non-extended sequence similarity to any known eukaryotic acyltransferase [55]. Since oleaginous rhodococci possess numerous DGAT isoenzymes with low substrate specificity, these bacteria could be considered valuable sources of enzymes potentially applicable to the production of a variety of lipids through genetic engineering strategies or synthetic biology.

Compared with other oleaginous microorganisms, rhodococci can be considered robust biofactories for the production of microbial oils. They possess similar characteristics to other oleaginous models, but with some advantages mainly based on their metabolic capacity to degrade a wide range of chemical compounds (see below), their high tolerance to different types of stresses and cultivation conditions, and their amenability to genetic and metabolic manipulation, among other features. The biological properties of rhodococci shared with oleaginous microalgae, yeasts, and fungi make them commercially interesting. However, the large-scale industrialization of microbial oils is still not possible due to high production costs. It is necessary to promote some innovations of fermentation technologies and to develop efficient engineering of oleaginous strains to improve the competitiveness of microbial lipid production.

## 4. Catabolism of Different Carbon Sources and Lipogenesis Pathways Are Coupled in Oleaginous *Rhodococci*

**Figure 2:**
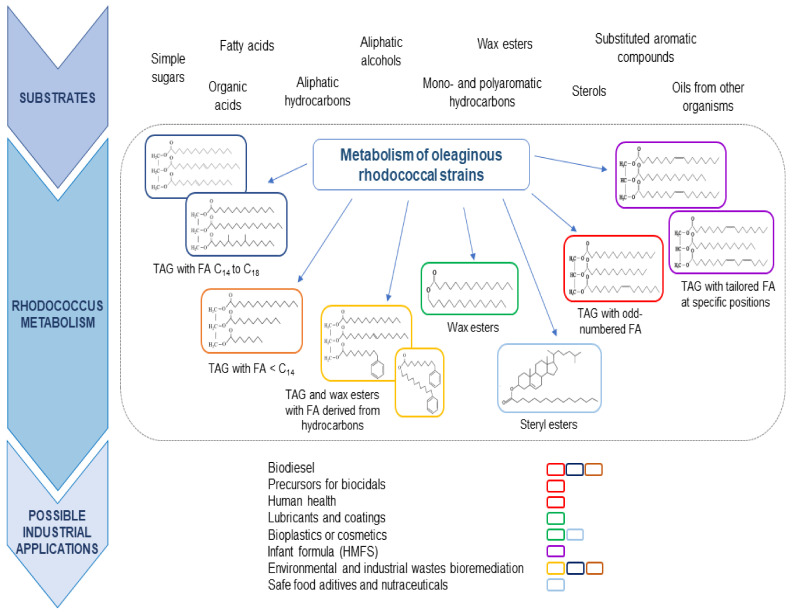
Biotransformation of multiple carbon and energy sources by oleaginous *Rhodococcus* strains to industrially relevant TAG.

As mentioned above, oleaginous rhodococci are equipped with a complete and redundant set of genes/proteins for the conversion of key metabolic intermediates to TAG. This is a basic requirement to consider a microbial system as a biological platform for the production of SCO. However, the additional property that supports the potential use of rhodococci as biofactories for the production of microbial oils is their enormous capacity to degrade a wide variety of substrates that are utilized for the production of metabolic intermediates available for lipogenesis pathways. Thus, rhodococci possess the natural ability to efficiently couple catabolism and anabolism during cell growth on carbon sources with different chemical structures for the synthesis of significant amounts of lipids. The high flexibility and robustness of its metabolism define its ability to oxidize substrates for the production of a wide diversity of precursors for lipid biosynthesis, including a variety of unusual fatty acids that can be incorporated in the accumulated lipids (Figure 2). In this section, we will include the most representative carbon sources that support good growth and lipid synthesis and accumulation by oleaginous rhodococci. The use of lignocellulosic substrates for rhodococcal lipid biosynthesis has been very well-covered in recently published reviews [56,57,58]. Thus, the focus of this section goes beyond this feedstock.

### 4.1. Sugars and Organic Acids as Carbon Sources

In general, oleaginous rhodococci accumulate TAG but not WE during the cultivation of cells on glucose or gluconate [8,9]. Under nitrogen-limiting conditions, which promote lipid accumulation, *R. opacus* and *R. jostii* oxidize glucose through ED and PP pathways to yield metabolic intermediates and reduced equivalents for lipogenesis [11,12,59]. In general, these substrates support good growth and lipid accumulation by oleaginous rhodococci, reaching between 50–75% of TAG (of CDW) [8]. Fed-batch fermentations using sugar beet molasses and sucrose as sole carbon sources yielded high-density cultures of *R. opacus* PD630 (37.5 g cell dry matter CDM per liter) with a TAG content in the cells of 52% at the 30 liter scale stirred tank bioreactor [60].

Usually, the metabolic intermediates generated by sugars or organic acid catabolism are used for de novo biosynthesis of saturated and unsaturated straight chain-length fatty acids (14 to 18 carbon atoms), with palmitic and oleic acids as main products [8,9,16]. Thus, SCO from oleaginous rhodococci can be considered promising feedstock for biodiesel production because of their similar fatty acid composition to vegetable oils [61]. Curiously, an unusually high fraction of fatty acids with an odd number of carbon atoms occurs in the accumulated TAG during the cultivation of cells on sugars or organic acid as sole carbon sources [62,63]. The amounts of odd-numbered fatty acids depend on the substrates used as carbon sources. Substrates as propionic and valeric acids stimulate the production of odd-numbered fatty acid-enriched TAG by rhodococci [62]. The TCA cycle and the methylmalonyl-CoA pathways are the main sources of the key precursor (propionyl-CoA) for the synthesis of odd-numbered fatty acids in rhodococci [62]. In general, odd-numbered fatty acids are found as minor constituents in many bacteria [64,65]. For this reason, the ability of the oleaginous rhodococcal strains to synthesize and incorporate this type of fatty acid into TAG is an important differential property in relation to other SCO-producing microorganisms, which naturally produce negligible amounts of odd chain fatty acids, such as *Yarrowia lipolytica*, among others [65]. Odd chain-length fatty acids are considered valuable products for their applications in the medical, therapeutic, and nutritional industries, as well as in chemical industries as precursors for manufacturing agricultural chemicals such as biocides, flavor and fragrance intermediates, hydraulic fluids, plasticizers, and coatings, among other applications [63,65,66]. Thus, oleaginous rhodococci can be considered a biological source of valuable oils, which are not readily obtainable via traditional petrochemical processes, with wide industrial applications.

### 4.2. Aliphatic Hydrocarbons

Oleaginous rhodococci are able to utilize aliphatic hydrocarbons (C_8_–C_16_) for growth and lipid accumulation [39,67]. They usually degrade alkanes by sequential oxidation reactions to alcohols, aldehydes, and finally fatty acids. Fatty acids can be either oxidized by β-oxidation pathway to enter in the TCA cycle, or directly incorporated to TAG in rhodococcal cells [62]. These results demonstrated that rhodococcal cells are able to utilize pre-formed fatty acids as precursors for SCO production. *R. opacus* PD630 and *R. jostii* RHA1 were able to accumulate between 28% to 39% (of CDW) of TAG during the cultivation of cells on *n*-alkanes (from C_15_ to C_18_), with fatty acids directly related to the carbon skeletons of hydrocarbons [8,9,62]. Castro et al. reported the accumulation of TAG by *R. opacus* B4 in comparison to *R. opacus*PD630, using different carbon sources for cell cultivation [39]. When they used hexadecane as a sole carbon source, strain B4 produced 0.09 and 0.14 g/L of TAG at 24 and 72 h, while PD630 only accumulated 0.05 and 0.04 g/L. In addition, *R. opacus* B4 showed a higher variability in fatty acid composition than *R. opacus* PD630, when both strains were cultivated on hexadecane [39]. These results highlight the variability between species/strains in relation to the conversion of aliphatic hydrocarbons to SCO, and their potential for environmental remediation coupled to the production of value-added compounds.

### 4.3. Alcohols

Only few studies have been focused on the potential use of alcohols for SCO production by oleaginous rhodococci. Chu et al. have recently found that *R. opacus* PD630 is able to produce up to 48% of CDW of oil enriched in odd-numbered fatty acids from 1-propanol and glucose as carbon and energy sources [68]. In addition, the amount of accumulated TAG reached up to 55% of CDW after increasing 1-propanol concentration in the cell cultures [63]. Transcriptomic analyses revealed the redirecting of carbon flow from the methylmalonyl-CoA pathway toward the generation of propionyl-CoA, which is used for de novo odd-numbered fatty acids biosynthesis [63]. Kosa and Ragauskas reported that *R. opacus* DSM1069 is able to use coniferyl alcohol as a carbon source and synthesize TAG from it [69]. The utilization of phenol by *R. opacus* for the production of TAG will be discussed in the next sub-section.

Glycerol, generated as a by-product during the biodiesel production process, is an attractive substrate for SCO synthesis. Oleaginous rhododocci such as *R. opacus* PD630 and *R. jostii* RHA1 exhibit a prolonged lag phase of several days before growing during the cultivation of cells on glycerol as a sole carbon source [70]. For this reason, Kurosawa et al. applied a self-adaptive evolution strategy to improve the production of TAG from glycerol using the engineered *R. opacus* strain MITXM-61 [71]. The evolved strain was able to grow on a high concentration of glycerol, reaching 40.4% of CDW of TAG. In another study, the heterologous expression of two glycerol-catabolic genes (*glpK1D1*) from *R. fascians* significantly reduced the extension of the lag phase and improved glycerol consumption and TAG production by *R. opacus* PD630 (app. 41% TAG of CDW) [70]. Interestingly, *R. fascians* F7 exhibited an oleaginous phenotype during growth on glycerol, showing fast growth and lipid accumulation (up to 44.6% TAG of CDW). These results highlight the metabolic versatility exhibited by rhodococci for producing SCO from glycerol.

Recently, Holert et al. described the production of steryl esters after cultivation on steroid compounds under nitrogen-limiting conditions [72]. Under these conditions, *R. jostii* RHA1 accumulated TAG and phytosteryl/cholesteryl esters from phytosterol and cholesterol. Cholesteryl esters reached up to 7% of CDW and represented 2/3 of lipids in cell vacuoles [72]. These new products co-produced by oleaginous rhodococci in addition to TAG, are also industrially interesting to provide functional foods, nutraceuticals or cosmetics.

### 4.4. Aromatic Compounds

Members of *Rhodococcus* genus possess a wide genetic and enzymatic repertoire for the degradation of aromatic compounds [73,74,75]. These substances support not only good growth but also significant lipid accumulation by oleaginous rhodococci. *R. jostii* RHA1 was able to produce up to 55% of TAG (CDW) during growth on benzoate as the sole carbon source under nitrogen-limiting conditions [16]. Palmitic acid (C_16:0_) and heptadecanoic acid (C_17:0_) were the predominant fatty acids produced during the cultivation of cells on benzoate. The main genes expressed in RHA1 in that study were those involved in benzoate catabolism, ammonium assimilation, the methylmalonyl-CoA pathway, and fatty acid and TAG biosynthesis, as revealed transcriptomic analyses of cells grown under conditions of N-limitation and N-excess [16].

Roell et al. reported the ability of *R. opacus* PD630 to use phenol, a lignin depolymerization model product, as a carbon source for growth and TAG biosynthesis [76]. This strain produced TAG from phenol with less unsaturated fatty acids and approximately 20% of branched chain C_18_ (BCFAs) [76,77]. The internal branches observed enhance cold-flow properties of a potential biodiesel, in comparison with those offered by terminal branches [78]. Using ^13^C-pathway tracing analyses, the authors demonstrated that phenol was metabolized mainly through the ortho-cleavage pathway, with TCA cycle and gluconeogenesis as key metabolic processes during growth on phenol and with NADPH generation mainly via NADPH-dependent isocitrate dehydrogenase [76]. Catabolite repression by other carbon substrates, such as glucose and acetate, was absent in *R. opacus* PD630, although benzoate inhibited phenol utilization. The authors suggested that the strong carbon fluxes through the TCA cycle in phenol-fed *R. opacus* make this host ideal for producing chemicals derived from TCA cycle intermediates, such as acetyl-CoA, which is the main precursor for lipogenesis [76]. In contrast, *R. opacus* was not a good model for the production of chemicals derived from sugar phosphates during growth on phenol since its carbon fluxes through gluconeogenesis are small [76].

Phenyldecane supported growth and accumulation of novel lipids in *R. opacus* PD630 during cultivation under nitrogen-limiting conditions [79]. The cultivation of cells on phenyldecane resulted in the formation of WE and TAG containing aromatic constituents derived from the substrate degradation (Figure 2). The hydrocarbon was degraded by monoterminal oxidation, followed by β-oxidation of the alkyl side-chain to phenylacetic acid and the oxidation of the latter to intermediates of the central metabolism. Phenyldecanoic acid, the monoterminal oxidation product, was used as a pre-formed precursor for the biosynthesis of a novel WE and TAG. The formation of the wax ester phenyldecyl-phenyldecanoate resulted from the condensation of phenyldecanoic acid and phenyldecanol, which were produced as metabolites during the catabolism of phenyldecane. In addition, two types of TAG were detected in phenyldecane-grown cells of *R. opacus* PD630: (i) TAG containing only odd- and even-numbered aliphatic fatty acids and (ii) TAG in which one fatty acid was replaced by a phenyldecanoic acid residue [79].

In another study, Goswami et al. analyzed the potential of *R. opacus* to utilize polycyclic hydrocarbons containing 2-, 3-, and 4- rings for growth and lipid accumulation [80]. They reported high ratios of degradation (72–79%) and lipid production (63–72% of CDW) by cells of *R. opacus* after 7 days of cultivation on naphthalene, phenanthrene, and fluoranthene. The fatty acid profiles of the accumulated TAG were mainly composed of even-numbered saturated and unsaturated fatty acids with C_8_ to C_24_, a composition with good potential for biodiesel production [80].

Silva et al. described the isolation from a hydrocarbon-polluted soil sample of a bacterial strain identified as *R. jostii* 602. This native strain was able to produce TAG during cultivation on naphthalene and naphthyl-1-dodecanoate. Interestingly, TAG produced by resting cells of *R. jostii* 602 in the presence of naphthyl-1-dodecanoate contained only short-chain-length fatty acids (from C_8_ to C_12_), suggesting an initial attack of the substrate by an esterase releasing 1-naphthol and dodecanoic acid, which was subsequently degraded by β-oxidation [25].

These studies demonstrate the wide metabolic repertoire of oleaginous rhodococci for the degradation of aromatic compounds and their good potential for converting these substances into lipids, such as TAG and WE, with a diversity of chemical structures (Figure 2).

## 5. Conversion of Industrial Wastes into Microbial Oils by *Rhodococci*

Diverse studies demonstrated that oleaginous rhodococci could accumulate intracellular lipids through cultivation on various industrial wastes. These microorganisms are able to produce different amounts of bacterial oil depending on the substrate or growing conditions (Table 2). Thus, oleaginous rhodococci may represent a valuable source of alternative oils that may possess similar chemical composition to that of the most commonly used vegetable oils in the biofuel or oleochemical industries. Table 2 presents microbial lipid contents and cellular biomass production after the cultivation of various rhodococcal strains on different industrial wastes.

Strains belonging to *R. opacus* exhibited good capability for growing and producing lipids from whey, which is a waste of the dairy industry generated worldwide in enormous quantities (Table 2). Interestingly, strains belonging to *R. jostii*, other oleaginous rhodococcal species, were not able to grow on whey because some genes coding for transport systems (LacEFGK) and the β-galactosidase (LacB) enzyme for lactose cleavage are lacking in this species [81]. *R. opacus* is a robust candidate for SCO production from whey because this species possesses a complete genetic endowment for degrading lactose and galactose, main constituents of whey, as well as for lipid biosynthesis from these substrates.

Gouda et al. described the production of SCO by *R. opacus* PD630 from food sugar-rich wastes, such as sugar cane molasses, carob, and orange wastes and sweet whey (Table 2) [82]. Fatty acid profiles in TAG were highly affected by the carbon source. In the presence of sugar cane molasses, apple pomace, and potato infusion, the even-numbered fatty acid fraction was dominant in cells, whereas odd-numbered fatty acids predominated during the cultivation of *R. opacus* PD630 on carob wastes as a carbon source [82]. According to the obtained results, the authors concluded that agro-industrial residual materials, especially orange waste, can be considered inexpensive feedstocks for SCO production by *R. opacus*. In addition, the variability in fatty acid composition of lipids with different carbon sources provides the chance to produce oils with different properties. Other studies demonstrated the ability of *R. opacus* to produce high-cell-density cultures from sugar-rich wastes at different scales. In this context, Kurosawa et al. proposed that *R. opacus* PD630 possesses great potential to produce oils for the biodiesel industry using starchy cellulosic feedstocks as carbon sources. They reported the high-cell-density cultivation of strain PD630 using high glucose concentrations (240 g/L for TAG production [38]). In another study, Voss and Steinbüchel optimized fed-batch fermentations of *R. opacus* PD630 at the 30-L scale in a stirred tank bioreactor containing sugar beet molasses and sucrose as sole carbon sources, resulting in the production of 37.4 g of cell dry matter (CDM) per liter with a TAG content in the cells of 52% (CDW). In addition, the authors performed a high-cell-density cultivation at a 500L pilot-plant scale, yielding high cell densities and high concentrations of TAG in the cells (Table 2) [60]. They proposed that the cultivation of *R. opacus* PD630 on agricultural products, such as beet molasses, can be applied to the biotechnological production of SCO and related products. Saisriyoot et al. also demonstrated that sugar cane molasses could be effectively used to produce biomass and lipids by *R. opacus* PD630 in a wide range of osmotic conditions (Table 2) [83].

Le et al. analyzed the bioconversion of second-generation cellulosic ethanol waste streams into SCO via oleaginous bacteria. They used one- and two-stage alkali/alkali-peroxide pretreatment waste streams of corn stover as feedstocks in 96 h batch reactor fermentations with *R. opacus* PD630, *R. opacus* DSM 1069, and *R. jostii* DSM 44719. The best performance was reached by *R. opacus* PD630, which converted 6.2% of organic content with a maximal total lipid production of 1.3 g/L and accumulated 42.1% in oils based on cell dry weight after 48 h [84]. The authors concluded that the use of industrial effluents that contain a fraction of sugars and lignin to produce cellular biomass and oils by rhodococci could be optimized, coupling fermentation procedures with genetic engineering on oleaginous bacterial strains. He et al. used a co-fermentation strategy to convert dilute alkali corn stoven lignin to SCO using a wild-type and an engineered rhodococcal strain, such as *R. opacus* PD630 and *R. jostii* Van A^-^ (Table 2). Co-fermentation with both strains produced higher amounts of lipids than single strain cultivation. Palmitic acid and oleic acid were the main constituents of accumulated lipids during the cultivation of cells on lignin as a sole carbon source [85]. This study demonstrated that rhodococcal strains possess the necessary pathways to degrade lignin to aromatic compounds, which can be oxidized via the TCA cycle, generating precursors for lipogenesis. The authors indicated that the conversion of lignin to lipids can be improved by applying engineering approaches to rhodococcal strains.

A variety of industrial effluents have been considered as carbon sources for SCO production by oleaginous rhodococci. Paul et al. reported the application of *R. opacus* for simultaneous raw refinery wastewater treatment and production of SCO. They applied different strategies for cell cultivation, including batch, fed-batch, sequential batch, continuous, and continuous with cell recycling using a low-cost tubular ceramic membrane (Table 2). The authors concluded that *R. opacus* serves as a good source of oils during wastewater treatment, with excellent biofuel properties [86]. Similarly, Kumar et al. reported the production of SCO by *R. opacus* with good potential for the biodiesel industry using raw dairy wastewater as a carbon source for cell cultivation. The accumulated lipids from this residual substrate contained more saturated than unsaturated fatty acids. The biodiesel produced by either ex-situ or in-situ transesterification of oils synthesized by *R. opacus* revealed the presence of methyl palmitate (34.90%), methyl stearate (35.48%), methyl myristate (29.79%), methyl linoleate (27.87%), and methyl palmitate (25.85%) as the main esters [87]. In another study, Gupta et al. proposed that *R. opacus* PD630 can be successfully scaled up for the efficient conversion of dairy wastewater to SCO with application for biodiesel production. They cultivated *R. opacus* cells in a bioreactor operated under three different modes: fed-batch, continuous, and continuous cell recycling. Maximum lipid accumulation was obtained when the bioreactor was operated under the continuous cell recycling mode (Table 2) [88]. *R. opacus* was also able to utilize biomass gasification wastewater for lipid accumulation with good potential for biodiesel production (Table 2) [89].

Oleaginous rhodococci are able to produce SCO from waste materials with complex chemical composition. Pyrolysis of biomass, such as softwoods, hardwoods, and grasses, is a technical procedure to utilize lignocellulosic resources for the production of chemicals and other products, which involves a thermal decomposition process at elevated temperatures (i.e., 400–600 °C) under an inert environment. Light oil is a liquid residual product from pyrolysis that contains large quantities of water (at least 40%) with variable amounts of organic compounds including various acids, methanol, and some aromatic-structured compounds [90]. *R. opacus* PD630 and *R. opacus* DSM 1069 were able to use pyrolysis light oil for growth and lipid accumulation (25.8% and 22.0% of CDW, respectively), with palmitic acid and stearic acid as the main fatty acid residues [90]. The authors proposed pyrolysis light oil as an additional feedstock for biodiesel production using *R. opacus* strains as an oleaginous source of SCO.

Glycerol is an interesting resource for SCO production due to its low cost and its increasing availability as a by-product of the biodiesel industry and alcoholic beverage production processes. Different rhodococcal species exhibited a differential ability to grow and produce lipids from glycerol. *R. fascians* and *R. erythropolis* grow fast and produce significant amounts of TAG from glycerol, whereas *R. opacus* and *R. jostii* exhibit a prolonged lag phase of several days before growing and accumulating lipids [70]. However, the use of the canonical oleaginous rhodococci, such as *R. opacus* or *R. jostii*, to convert glycerol into SCO could be significantly improved by genetic manipulation of strains, including adaptive evolution procedures [71] or the heterologous expression of glycerol-catabolic genes (e.g., *glpK* and *glpD*) [70]. These results demonstrate that according to the rhodococcal species/strains, the metabolic responses could be different depending on their respective genetic repertoire in relation to the employed carbon source.

Another source of carbon for the production of lipids is the olive mill waste (OMW), which is a residual material obtained during the production of olive oil. This industry represents an important economic activity in South America, Europe, and other regions in the world. It has been estimated that 10 million m^3^ of this liquid effluent are generated per year, which corresponds to an equivalent load of the wastewater generated from about 20 million people [93,94]. Oleaginous rhodococci, such as *R. opacus, R. wratislaviensis,* and *R. jostii,* were more efficient at producing cell biomass (2.2–2.7 g/L) and lipids (77–83% of CDW, 1.8–2.2 g/L) from OMW in comparison to other rhodococcal species [43]. The efficiency of the bioconversion process could be significantly enhanced by the overexpression of a gene coding for a fatty acid importer (Ltp1) in *R. jostii* RHA1, which promoted a 2.2-fold increase in cellular biomass value with a concomitant increase in lipids production during the cultivation of cells in OMW [23]. This study demonstrated that the bioconversion of OMW to microbial lipids is feasible using more robust rhodococal strains.

Several million tonnes of municipal solid waste (MSW) are annually produced worldwide. The organic fraction of MSW (OMSW) typically comprises approximately 50% lignocellulose-rich material. Dornau et al. analyzed the potential of eight biotechnologically useful microorganisms (*Clostridium saccharoperbutylacetonicum*, *Escherichia coli*, *Geobacillus thermoglucosidasius*, *Pseudomonas putida*, *Rhodococcus opacus*, *Saccharomyces cerevisiae*, *Schizosaccharomyces pombe*, and *Zymomonas mobilis*) to grow and produce diverse bioproducts from OMSW. Enzymatic hydrolysate of OMSW fiber contained high concentrations of glucose (5.5%, *w*/*v*) and xylose (1.8%, *w*/*v*) but was deficient in nitrogen and phosphate [91]. Interestingly, three species (*Z. mobilis*, *S. cerevisiae,* and *R. opacus*) became more efficient during OMSW fiber hydrolysate fermentation. *R. opacus* MITXM-61 produced TAG to 72% of the maximum theoretical fermentation yield and could theoretically produce 91 kg of TAG per tonne of OMSW [91]. *R. opacus* MITXM-61 is an engineered strain [95] with the ability to simultaneously utilize D-glucose and D-xylose in lignocellulosic hydrolysates. The TAG produced by *R. opacus* grown on OMSW fiber exhibited the typical fatty acid composition of this species, including C_14_–C_18_ fatty acyl-residues with an abundance of palmitic acid. This study demonstrated that *R. opacus* is well-suited for growth and lipid production on OMSW fiber hydrolysate and that this residual material is another potential feedstock for producing biofuels and chemicals [91].

Saisriyoot et al. cultivated *R. opacus* PD630 cells on wastewater of a petroleum processing facility supplemented with molasses and ammonium chloride to produce cellular biomass and microbial oils (Table 2). The authors concluded that the accumulated lipids could replace vegetable oils for biodiesel production [92].

These results demonstrated that *R. opacus* and other oleaginous rhodococcal species are promising candidates for developing microbial biofactories for oil production using a diversity of industrial wastes as inexpensive feedstocks. This novel technology would allow the diversification of the production field of oils for the industry and at the same time reduce the impact of environmental pollution through the usual waste management practices.

Cell harvesting and oil extraction and recovery are critical downstream processes to be considered for evaluating the cost and feasibility of SCO production from diverse industrial wastes. Different methods have been applied to improve yields of oil harvests from oleaginous microorganisms and to reduce costs of the process [96]. Particularly in rhodococci, as well as in other lipid-accumulating bacteria, solvent extraction has been the most common method used for lipid purification. However, this methodology utilizes toxic, flammable, and volatile solvents, such as methanol, chloroform, or hexane, and also needs an additional step to separate lipids from solvents. In general, to overcome drawbacks associated with solvent extraction, different strategies have been applied to oleaginous microorganisms integrating solvent extraction with mechanical processes. However, all of them usually demand high-cost technologies [97]. Recently, innovative methods based on the application of lytic enzymes or bacteriophages have been developed to enable cell disruption and release of intracellular components, including the accumulated oils [96,97,98]. In this context, phage-based lipid extraction has been applied to *R. opacus* to release accumulated lipids after programmed cell lysis. A tectiviral phage Toil was able to infect rhodococcal cells to release intracellular contents in the culture medium [98]. Approximately 30% of intracellular TAG could be recovered from the culture supernatants of Toil-infected *R. opacus* cells [98]. Although this technology seems to be promising to reduce overall production costs, more studies are needed to improve lipid recovery yields and to optimize the process since this technology seems to be affected by several factors that have to be controlled [98].

## 6. Engineering Approaches to Improve Lipid Accumulation in Oleaginous *Rhodococcus*

Different strategies have been applied to improve TAG accumulation in oleaginous microorganisms. These approaches involve modifications to cell culture conditions (type and quantity of carbon and nitrogen sources, temperature, pH, osmolarity, aeration, time, and continuous vs. batch cultures, among others) or genetic and metabolic engineering of the oleaginous microorganisms [99,100]. Engineering non-model microorganisms such as rhodococci possess some limitations due to the lack of a robust genetic toolbox in these species, in contrast to other oil-producing organisms such as yeasts [101,102].

Recently, new tools based on the CRISPR technology have been reported for genetic manipulation in *Rhodococcus* cells, expanding the possibilities to make improvements in these microorganisms [103,104]. Despite the limited existence of molecular tools for this genus, several efforts based on diverse genetic strategies have been applied in these bacteria to increase TAG production (and related lipids) under different growth conditions. Table 3 summarizes the main genetic modifications made in oleaginous rhodococci to specifically enhance lipid production.

One of the first genes targeted for improving TAG accumulation has been the *atf* gene coding for putative WS/DGAT enzymes. The main challenge of studying these enzymes is the high redundancy of WS/DGAT-coding genes occurring in oleaginous rhodococci, as well as the lack of information about their specific role in cells. In some rhodococcal strains, *atf* genes have been functionally characterized, and their roles in TAG biosynthesis and accumulation have been demonstrated [16,105,106]. In general, the overexpression of *atf* genes promoted an increase of approximately 10% (CDW) in TAG content in cells of oleaginous rhodococci. Phosphatidic acid phosphatase type 2 (PAP-2) (RO00075) is another enzyme of the Kennedy pathway that has been characterized in the oleaginous rhodococcal strain, *R. jostii* RHA1. Enzyme overexpression in strain RHA1 resulted in an increase inthe TAG content of approximately 10–15% (CDW) [111]. As occurs in other microorganisms, this enzyme seems to be important not only to supply DAG precursors for TAG biosynthesis but also as a key point for lipid synthesis regulation [114]. Until now, only few PAP-2 enzymes have been characterized in bacteria [115,116]. Although the Kennedy pathway is a key route for TAG biosynthesis in rhodococci, no other enzymes of this pathway have been characterized at the molecular level in these microorganisms.

In another study, Villalba and Alvarez reported a novel ATP-binding cassette transporter involved in long-chain fatty acid import and its role in TAG accumulation in *R. jostii* RHA1 [112]. Interestingly, the transporter coding gene (*ltp1*) is located adjacent to a cluster containing genes of the Kennedy pathway. Ltp1 overexpression led to an approximately six-fold and three-fold increase in cellular biomass and TAG production, respectively, when cells were cultivated on palmitic acid and oleic acid as carbon sources [112].

Fatty acid biosynthesis is a mandatory requirement for TAG biosynthesis. In oleaginous rhodococci, the FASI system is natively upregulated under nitrogen deprivation conditions that stimulate lipid accumulation and is a critical multifunctional enzyme that contributes to the fatty acyl-CoA pools [12,117]. To improve TAG biosynthesis in *R. opacus* PD630, Xie et al. overexpressed both the *fasI* operon and *atf2* gene for a higher carbon partition into lipid biosynthesis [109]. The recombinant strain exhibited a significant increase in lipid production from 21% (CDW) in the wild type to 55% (CDW) after overexpression of both genes during growth on 2% (*w*/*v*) of glucose as a sole carbon source [109].

Huang et al. analyzed the role of four native thioesterases in *R. opacus* PD630 and explored the overexpression of these enzymes to increase TAG production [107]. Thioesterases hydrolyze the thioester bond of acyl-ACPs for the production of fatty acyl-CoAs that are further converted to TAG and other lipids in cells [107]. Only two recombinants overexpressing native thioesterases had a significant effect on the total fatty acid contents, increasing wild-type strain PD630 by approximately 1.5 times and 20–30% (*w*/*w*) more than that of the control strain containing the empty inducible vector [107]. In addition, the contents of C17:1 and C18:1 fatty acids increased approximately between 20–35% compared with the control strain. The study demonstrated that engineering native thioesterases in *R. opacus* is an interesting strategy to improve lipid production and also tailor the composition of the fatty acid profile in cells.

Oleagenicity involves several reactions, pathways, and regulatory circuits for the appropriate distribution of metabolic resources in the cell. Central carbon metabolism provides energy, reducing equivalents, and contributes to the synthesis of intermediate compounds that act as precursors for storage lipid metabolism. For this reason, the manipulation of primary metabolism is a powerful tool to engineer SCO production in oleaginous rhodococci. The metabolic reactions responsible for the provision of primary precursors are bottlenecks in the transfer of metabolic flux from central to lipid metabolism and should be considered to increase the yield of microbial oils. In this context, MacEachran and Sinskey identified a gene in *R. opacus* PD630, referred to as *tadD*, encoding a non-phosphorylative glyceraldehyde 3-phosphate dehydrogenase enzyme that provides NADPH for fatty acid and TAG metabolism [13]. The authors postulated that TadD could have a key role during the transition from a vegetative growth phase to a lipid storage phase in this strain. The overexpression of TadD resulted in an increase in TAG (total fatty acids) accumulation from 17.59% (CDW) in the wild-type strain with the empty vector to 23.10% (CDW) in the recombinant strain [13]. On the other hand, Hernández and Alvarez demonstrated that the activities of NADP^+^-dependent malic enzymes (ME) contribute to TAG production in the oleaginous *R. jostii* RHA1 and *R. opacus* PD630 strains [14]. These enzymes catalyze the oxidative decarboxylation of malate to generate pyruvate, CO_2_, and NADPH, providing carbon precursors and reducing equivalents for lipogenesis. The overexpression of *RHA1_RS44255* gene encoding an NADP^+^-dependent ME in *R. jostii* RHA1 promoted an increase in total NADP^+^-ME activity and an up to 1.9-fold increase in total fatty acid production without sacrificing cellular biomass during the growth of cells on glucose as a sole carbon source [14]. The results of these studies confirmed that the manipulation of enzymes of central metabolism in oleaginous rhodococci constitutes a valuable strategy to increase bacterial oil production from different carbon sources.

TAG and other storage lipids are accumulated in the form of lipid droplets (LD) in rhodococci. Diverse studies have been conducted in these microorganisms to elucidate the ontogeny of LD and characterize their components, such as the associated phospholipids and proteins [113,117]. One of the most abundant proteins associated with LD in rhodococci is the protein known as TadA/MLDS, which seems to have a key role in the ontogeny of LD, in its structure and stability. The mutation and overexpression of this protein resulted in a decrease or increase in TAG content in cells, respectively, by affecting the size and number of the LD in cells [108,113]. However, at the moment the manipulation of proteins involved in the structural stability of LD in rhodococci does not seem to be an adequate strategy to increase the yields of oil production, since the knowledge about the dynamics of LD formation is scarce and difficult to control. How oleaginous rhodococci induce and regulate the formation of LD deserves further investigation and greater understanding.

TAG accumulation in oleaginous rhodococci is the result of the contributions of different sets of genes/enzymes probably regulated by different regulatory circuits that work in a highly coordinated manner. In this context, understanding the connection and regulation of the carbon flow between the central and lipid metabolism is a key factor for the development of oil-producing biofactories with improved traits. The composition of the multi-level regulatory network of oleaginous bacteria is surely complex, and our knowledge of the lipogenesis regulation is still incipient. Hernández et al. reported the identification of a transcriptional regulatory protein with a strong influence on lipid metabolism in *R. jostii* RHA1 and *R. opacus* PD630 [20]. The protein called NlpR (for **N**itrogen-**L**i**p**id-**R**egulator) is a global transcriptional regulator that responds to nitrogen deprivation and positively regulates both genes of nitrogen and lipid metabolisms, including *narK*, *nirD*, *fasI*, *plsC*, and *atf3* genes. ^13^C-labeling analysis demonstrated that NlpR regulator contributes to the oleaginous phenotype in *R. jostii* RHA1 to the allocation of carbon into the different lipid fractions in response to nitrogen levels, increasing the rate of carbon flux into lipid metabolism [21]. Interestingly, NlpR provides a stronger redirection of carbon flux toward lipogenesis and maximizes the capacity of cells to accumulate TAG under nitrogen starvation conditions; thus, this regulator can be considered as a potent lipogenic factor in oleaginous rhodococci. The *nlpR* overexpression resulted in an increase of 1.8- to 2-fold in total fatty acid content in *R. jostii* RHA1 pTipQC2/*nlpR* and *R. opacus* PD630 pTipQC2/*nlpR* strains grown on glucose in MSM1 medium (nitrogen-rich conditions) in comparison with their respective control cells (RHA1 pTipQC2 and PD630 pTipQC2) [20]. These results demonstrated that the overexpression of *nlpR* gene reinforces the carbon flux to fatty acid and TAG biosynthesis, during the cultivation of cells under nitrogen-rich conditions, which usually stimulate cell growth but not lipid accumulation. The manipulation of global regulatory proteins that influence lipid metabolism can be a powerful approach to enhancing lipogenesis in oleaginous rhodococci.

These actinobacteria are also used as biological platforms for the production of other lipids and derivatives for biotechnological purposes. In this context, Kim et al. successfully applied different engineering strategies to *R. opacus* strains to produce free fatty acids (FFAs), fatty acid ethyl esters (FAEEs), and long-chain hydrocarbons (LCHCs) [110].

According to results of these studies, it is clear that oleaginous rhodococci are non-model microorganisms amenable to genetic engineering to convert them into powerful biofactories for microbial oil production.

## 7. Wax Ester Biosynthesis in Oleaginous *Rhodococci*

Within neutral lipids, wax esters (WE) are valuable compounds for the cosmetic industry, and their microbial production could be used as an alternative to jojoba or sperm whale oil. One of its main advantages is the production of tailored WE from specific carbon sources and growth conditions.

**Figure 3:**
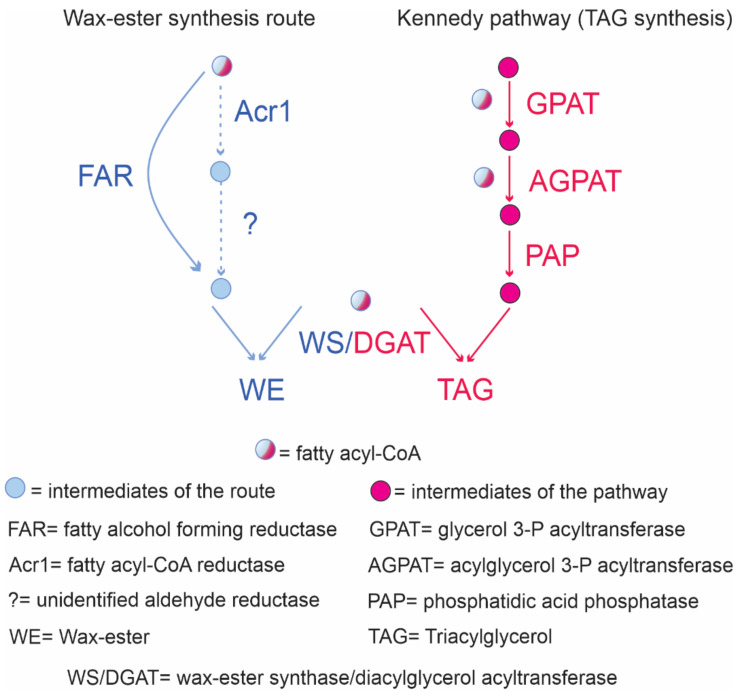
Overview of neutral lipid biosynthesis in bacteria. WE pathways are shown in blue while a scheme of the Kennedy route for TAG is shown in pink. Circles indicate compounds participating in each route. To give the fatty alcohol in WE synthesis, *Acinetobacter calcoaceticus* (dotted line) employs two steps. A second pathway is present in *Marinobacter aquaeolei* (continuous line) that in one step through a FAR gives the fatty alcohol. The final reaction involves the bifunctional WS/DGAT that utilizes a fatty acyl-CoA and diacylglycerol to form TAG.

WE production occurs in Gram-negative species such as *Acinetobacter baylyi*, *Acinetobacter calcoaceticus*, *Psychrobactercryohalolenti* K5, and *Marinobacter aquaeolei* VT8 [118,119] from preformed compounds or synthesized de novo from diverse carbon sources. WE biosynthesis in *Acinetobacter* sp. involves three enzymatic reactions [120]. First, an acyl-CoA is reduced by a NADPH acyl-CoA reductase, named Acr1, to its corresponding fatty aldehyde. Then, an unidentified NADPH fatty aldehyde reductase uses the fatty aldehyde as substrate synthesizing the corresponding fatty alcohol. Finally, the WE are formed by a fatty acyl-CoA connected to a fatty alcohol by an ester bond (Figure 3). In *Marinobacter aquaeolei* VT8, this pathway involves only two metabolic steps because its fatty acyl-CoA reductase is able to catalyze the four-electron reduction of the fatty acyl-CoA to the fatty alcohol [121]. The last step is similar in both strains. The reaction is catalyzed by a wax ester synthase (WS) that could also function as an acyl-CoA diacylglycerol acyltransferase (DGAT) for TAG synthesis. Thus, the metabolic pathways for both neutral lipids production are intertwined. They share not only a fatty acyl-CoA residue as a substrate but also the WS/DGAT enzyme catalyzing the final reaction (Figure 3).

Some Gram-positive strains belonging to Actinobacteria are also able to produce small amounts of WE when the fatty alcohol or the alkane backbone is supplied, for example, *R. opacus* PD630 grown on phenyldecane [79], hexadecane [9], or *Mycobacterium ratisbonense* SD4 grown on phytane [122].

*R. opacus* PD630 together with *R. jostii* RHA1 are the paradigm of TAG storage, and their ability to accumulate WE has never been characterized in detail. In this context, the detection of saturated WE by GC analysis was reported in one sample of wild-type *R. jostii* RHA1 grown in casamino acids under conditions favoring neutral lipid synthesis [123]. However, the authors indicated that their results were not consistent between samples. The detection of trace amounts of even-numbered WE in oleaginous *R. jostii* RHA1 grown on glucose was also reported by [124] in accordance with the results obtained by [123]. The metabolic pathway present in this strain was unclear until the authors, through a bioinformatic study of *R. jostii* RHA1 annotated genome, detected RHA1_RS30405 and RHA1_RS09420 [124]. The former showed a 43% identity with MAQU_2507, a fatty acyl-CoA reductase from *M. aquaeolei* VT8. The sequence was named FcrA, a 664 amino acid protein conserved in the *Rhodococcus* genus. RHA1_RS09420 exhibits a 46% identity with the Acr1 from *A. calcoaceticus*. Both genes are poorly expressed in the exponential and stationary phase under nitrogen-limiting conditions as indicated in RNA-seq analysis [124]. FcrA shows similarity to MAQU_2507, which exhibits the ability to completely reduce fatty acyl-CoA to fatty alcohols in one step. Thus, the authors cloned and overexpressed FcrA in *R. jostii* RHA1. The overproduction of FcrA in RHA1 resulted in a WE production representing 13% (CDW). WE composition showed that 20% were unsaturated and the acyl-CoA portion contained the double bond. In that work, *fcrA* was also deleted, resulting in WE levels similar to the wild-type strain. To optimize WE production, *fcrA* was expressed under the control of different strong constitutive promoters, yielding a WE maximum of 15% (CDW) [125]. *R. opacus* PD630 is also a prominent host for synthetic production of WE by the heterologous expression of MAQU_2220, another fatty acyl-CoA reductase present in *M. aquaeolei* VT8 [126]. MAQU_2220 was identified and functionally characterized as a FAR catalyzing the 4-electron reduction of fatty acyl-CoA and fatty acyl-ACP to fatty alcohol, after its heterologous expression in *E. coli* [127]. Considering the metabolic pathway that drives WE synthesis and its connection with TAG production (Figure 3), FAR activity may be the bottleneck in oleaginous rhodococci as strain PD630. Thus, the expression of an already characterized enzyme able to produce the desired fatty alcohols has allowed the rewiring of rhodococcal metabolism to produce high titers of WE. The authors reported the production of WE by recombinant *R. opacus* from different substrates ranging from structurally related substrates (hexadecane) to industrial dairy wastes (whey) [126]. Furthermore, the engineered strain was able to synthesize WE de novo from gluconate as a carbon source with WE reaching more than 45% of CDW. In general, these results serve as an interesting proof of concept, demonstrating the adaptability of the metabolic chassis of oleaginous rhodococci to the incorporation of novel biochemical reactions dealing with the production of novel lipids.

## 8. Conclusions

Available information indicated that oleaginous rhodococci are important components of bacterial communities that actively participate in the recycling dynamics of matter in a variety of natural environments. The repertoire of rhodococcal genes, which includes an extraordinary diversity of genes devoted to the degradation of a wide range of organic compounds, and numerous genes involved in lipid metabolism are key properties that promote their survival and persistence in various environments. For many years, rhodococci have received much attention from the scientific community to study their role and potential application in environmental technologies for cleaning and recovering polluted environments. However, its outstanding ability to efficiently couple catabolism and anabolism to produce significant amounts of TAG as storage lipids expanded the study perspectives toward the microbial oil production field. This unique feature of oleaginous rhodococci is probably supported by multiple layer physiological and molecular mechanisms that rely on extensive numbers of regulatory factors within integrated and complex signal processing pathways. In the last years, many efforts have been devoted to decipher the oleaginous nature of rhodococci and to know what are the components that define the lipogenic machinery in these microorganisms. Although our fundamental knowledge on rhodococcal oleagenicity is still fragmentary, the available studies included in this review have shed some light on the physiological and molecular mechanisms involved in the process. The application of global study strategies is contributing to generate a holistic vision on the cellular process. Beyond the scientific interest in elucidating basic aspects of the oleagenicity process in these bacteria, recently there has been a growing interest in the technological perspective to apply these bacteria for the conversion of industrial waste into SCO. Recent studies on the technological capabilities of oleaginous rhodococci to convert diverse waste materials into SCO highlighted the good potential of these bacteria to be applied as microbial biofactories for oil production at a large industrial scale. In addition, diverse molecular approaches devoted to produce engineered rhodococcal strains with increased productivity of oils or expanded capabilities for utilizing wastes as source of oils have demonstrated that rhodococci are amenable to genetic and metabolic improvements. The design of new tools based on the CRISPR technology for genetic manipulation in rhodococci allows foreseeing a rapid advance both in the generation of basic knowledge on its biology and in the development of new, improved, and adapted strains to establish specific technological oil production processes using different types of industrial wastes. In turn, the growing interest in using these microorganisms competitively as oil biofactories will significantly boost the production of new scientific knowledge on the mechanisms involved in this process.

We think that oleaginous rhodococci, such as *R. opacus* or *R. jostii*, can be considered promising oil-producing biofactories, expanding the repertoire of the canonical microbial oil-producers, such as oleaginous yeasts, fungi, and microalgae. As mentioned in this review, each oil-producing microbial model exhibits advantages and disadvantages that must be adapted to each technology to be developed. The potential of these oleaginous rhodococcal species as SCO sources is supported by their evolutionarily specialized metabolism, affording an awesome capacity for biocatalytic transformations. Previous studies demonstrated that some strains such as *R. opacus* PD630 and *R. jostii* RHA1 are well-characterized native oil producers that can be utilized as “chassis” or heterologous hosts to facilitate the yield optimization of lipid compounds of industrial interest. Moreover, they are suitable for large-scale bioreactor fermentation at least from glucose and sugar derivatives and are amenable to genetically reprogram cellular metabolism. All these results reflect the emergent role of these rhodococcal strains as programmable biochemical workhorses, providing promising platforms for sustainable production of diverse lipid-derived products from different agroindustrial wastes. Despite the strengths mentioned, some limitations and bottlenecks in these microorganisms should also be taken into account in order to consider them efficient oil biofactories at an industrial scale. The genetic manipulation of these actinobacteria is more difficult compared to other bacterial species, such as *Escherichia coli* or *Coynebacterium glutamicum*, because the genomes of oleaginous rhocococci are often larger and more complicated in structure and due to the lack of efficient genome editing tools available to them. The combination of mutant and recombinant screening with omics will help to identify new targets and strategies to engineer strains for production improvement. The application of genome-scale metabolic models can be useful to predict genes and pathways involved in the metabolic flux to a target bioproduct for rationally improving the process of interest. In this context, the increasing availability of high-quality omics data from oleaginous rhodococci will provide a better scenario for developing new omics-based engineering strategies. The construction of integrated (multi)-omics databases including different rhodococcal and actinobacteria species could also promote the development of programmed biofactories for the production of oils from different industrial wastes. Major efforts should be devoted to the optimization of bioreactor fermentations and downstream processes to achieve maximum yield and ensure the recovery of high-quality SCO from rhodococci. Finally, the discovery of new rhodococcal strains with the ability to produce oils could be of interest in this field for using them as a new chassis or as a source of heterologous genes to increase the productivity of well-characterized strains.

Hopefully, this review will stimulate further research on these fascinating microorganisms, covering open fundamental questions and technological gaps that allow these microbial biofactories to be successfully applied at an industrial scale.

## Figures and Tables

**Table 1:** Comparison between oleaginous rhodococci and other representative oleaginous single-cell microorganisms (SCMs).

SCM Type	Microorganisms	^a^ Cultivation Mode	Cellular Biomass	^b^ Total Fatty Acid Content (% CDW) and Cell Culture Time	^c^ Main Fatty Acid Composition	References
Actinobacteria (oleaginous rhodococci)	*R. jostii* RHA1	Shake flask	-	50–60%, 2 days	SFAs (**C_16:0_**, C_17:0_); MUFAs (C_16:1_, C_17:1_, C_18:1_)	[9]
	*R. opacus* PD630	-Shake flask -Batch bioreactor -Shake flask	- 77.6 g/L 3.9 g/L	50–76%, 2 days 38%, 4 days 28.7%, 1 day	SFAs (**C_16:0_**, C_17:0_); MUFAs (C_17:1_, C_18:1_)	[8,23,24]
	*R. jostii* 602	Shake flask	-	60–70%, 2 days	SFAs (**C_16:0_**, C_17:0_, C_18:0_); MUFAs (C_18:1_)	[25]
	*R. opacus* B4	Shake flask	2.5 g/L	23%, 1 day	SFAs (**C_16:0_**, C_17:0_); MUFAs (C_17:1_, C_18:1_)	[24]
	*R. rhodochrous*	Batch bioreactor	7 g/L	43%, 7 days	SFAs (C_16:0_); MUFAs (**C_18:1_**)	[26]
	*Rhodococcus sp.* YHY01(marine strain)	Shake Flask	1.3 g/L	56%, 3 days	SFAs (**C_14:0_**, C_16:0_, C_18:0_); MUFAs (C_18:1_)	[27]
Yeast	*Yarrowia lipolytica* ACA-DC50109	Continuous bioreactor	12.2 g/L	47.5%, 9 days	SFAs (C_16:0_); MUFAs (C_16:1_,**C_18:1_**, C_18:2_)	[28]
	*Rhodosporidium toruloides* Y4	Fed-Batch bioreactor	37.2 g/L	64.5%, 6 days	SFAs (C_16:0_); MUFAs (**C_18:1_**, C_18:2_)	[29]
	*Lipomyces starkeyi*NBRC10381	Shake flask	21.6 g/L	71.5%, 6 days	SFAs (C_16:0_); MUFAs (**C_18:1_**)	[30]
	*Rhodotorula glutinis*	Shake flask	6.8 g/L	43.0%, 4 days hs	SFAs (C_16:0_); MUFAs (**C_18:1_**)	[31]
Microalgae (heterotrophic or mixotrophic conditions)	*Chlorella vulgaris* UTEX 259; UTEX 2714	Shake flask Shake flask	1.7g/L 6.1 g/L	21%, 6 days 37.6%, 6 days	SFAs (**C_16:0_**); PUFAs (C_18:2_, GLA)	[32,33]
	*Chlamydomonareinhardtii*CC1010	Shake flask	0.37 g/L	47%	SFAs (C_16:0_); MUFAs (**C_18:1_**)	[34]
	*Neochloris oleoabundans* UTEX 1185	Batch bioreactor	9.2 g/L	52%, 9 days	SFAs (C_16:0_, C_18:0_); MUFAs (**C_18:1_**, C_18:2_)	[35]
Fungi	*Mucor circinelloides*WJ11	Batch bioreactor	14 g/L	36%,3.5 days	SFAs (C_16:0_); MUFAs (**C_18:1_**); PUFAs (C_18:2_, GLA)	[36]
	*Mortierella alpina* ATCC 32222	-Shake flask -Batch bioreactor	18 g/L 5 g/L	18%, 6 days 20%, 3 days	SFAs (C_16:0_, C_18:0_); MUFAs (C_18:1_); PUFAs (**ARA**)	[37,38]
	*Mortierella isabellina* ATHUM 2935	Shake flask	10.2 g/L	83%, 4 days	SFAs (C_16:0_); MUFAs (**C_18:1_**); PUFAs (C_18:2_)	[39]
	*Cunningha mellaechinulata* FR3	Shake flask	8.6 g/L	34%, 8 days	SFAs (C_16:0_); MUFAs (**C_18:1_**); PUFAs (C_18:2_, GLA)	[40]
	*Aspergillus oryzae* BCC14614	Shake flask	16 g/L	12–20%, 3–5 days	SFAs (C_16:0_; C_18:0_); MUFAs (C_18:1_); PUFAs (**C_18:2_**)	[41,42]

^a^ For a better comparison, all data were based on strains that were grown under a high C/N ratio conditions with glucose as the sole carbon source. ^b^ Refers to total fatty acid content in parental strains without any genetic modification for lipid content improvement. ^c^ Only fatty acids greater than 10% were considered. The highest component is highlighted in **bold**. References. PUFAs: polyunsaturated fatty acids; MUFAs: monounsaturated fatty acids; SFAs: saturated fatty acids; ARA: arachidonic acid; GLA: γ-linolenic acid.

**Table 2:** Bioconversion of different feedstocks into oils by *Rhodococcus* strains.

Strains	Wastes	Lipid Content (% *w*/*w* of CDW)	Biomass	References
*R. opacus* PD630	Whey permeate	45.1	6.1 ^a^	[81]
*R. opacus* MR22	Whey permeate	46.1	6.3 ^a^	[81]
*R. opacus* PD630	Sugar cane molasses	96	NR	[82]
*R. opacus* PD630	Orange waste	83	NR	[82]
*R. opacus* PD630	Sweet whey	82	NR	[82]
*R. opacus* PD630	Beet molasses and sucrose (Fed-batch biorreactor)	52	37.4 ^a^	[60]
*R. opacus* PD630	Beet molasses and sucrose (stirred tank reactor)	38.4	18.4 ^a^	[60]
*R. opacu*s PD630	Sugar cane molasses	29.8	11.9 ^a^	[83]
*R. opacus* PD630	Corn stover waste streams	42.1	2.13 ^a^	[84]
*R. opacuS* DSM 1069	Corn stover waste streams	12.6	2.02 ^a^	[84]
*R. jostii* DSM 44719	Corn stover waste streams	23.3	1.1 ^a^	[84]
*Co- fermentation R. opacus* PD630 and *R. jostii* RHA1VanA^-^	Alkali corn stover lignin	29	0.35 ^b^	[85]
*R. opacus* PD630	Raw refinery wastewater	86	3.6 ^a^	[86]
*R. opacus* DSM 43205	Raw dairy wastewater (Batch flask)	33.3	4.5 ^b^	[87]
*R. opacus* DSM 43205	Raw dairy wastewater (Batch bioreactor)	52	2.7 ^b^	[87]
*R. opacus* PD630	Dairy wastewater	79	4 ^a^	[88]
*R. opacus* DSM 43205	Biomass gasification wastewater	62.8	0.7 ^b^	[89]
*R. opacus* PD630	Light oil from pyrolysis biomass	25.8	0.82 ^a^	[90]
*R. opacus* DSM 1069	Light oil from pyrolysis biomass	22	0.90 ^a^	[90]
*R. opacus* PD630	Glycerol	38.4	3.8 ^a^	[70]
*R. opacus* MR22	Glycerol	35.5	3.3 ^a^	[70]
*R. jostii* RHA1	Glycerol	30.5	2.5 ^a^	[70]
*R. erythropolis* DSM 43060	Glycerol	38.3	4.1 ^a^	[70]
*R. fascians* F7	Glycerol	44.6	4.3 ^a^	[70]
*R. opacus* PD630-pTip-QC2/*glpK1D1*F7	Glycerol	41	4.4 ^a^	[70]
*R. opacus* MITXM-173	Glycerol	40.4	5.69 ^a^	[71]
*R. opacus* PD630	Olive oil mill	68.2	2.2 ^a^	[43]
*R. opacus* MR22	Olive oil mill	82.2	2.2 ^a^	[43]
*R. jostii* RHA1	Olive oil mill	67.3	2.6 ^a^	[43]
*R. wratislaviensis* V	Olive oil mill	88.4	2.6 ^a^	[43]
*R. opacus* MITXM-61	Organic fraction municipal solid waste fiber hydrolysate	48.9	32.7 ^a^	[91]
*R. opacus* PD630	Petroleum wastewater supplemented with molasses (Batch bioreactor)	52.5	5.91 ^a^	[92]
*R. opacus* PD630	Petroleum wastewater supplemented with molasses (Fed-batch bioreactor)	54.4	7.24 ^a^	[92]

^a^ Values of biomass expressed in g/L. ^b^ Values of biomass expressed by OD600 nm. NR not reported.

**Table 3:** Main genetic strategies applied in oleaginous *Rhodococcus* for TAG and other lipids improvement.

*Rhodococcus* Strain	Genetic Strategy	Goal of the Study	References
*R. opacus* PD630	Disruption and overexpression of *atf1, atf2* genes	Gene role evaluation in TAG biosynthesis. TAG accumulation improving	[105,106]
*R. opacus* PD630	Disruption and overexpression of *nlpR* gene	Gene role evaluation in TAG biosynthesis. TAG accumulation improving, under nitrogen rich-conditions	[20]
*R. opacus* PD630	Thioesterasesoverexpression	Fatty acid (FA) biosynthesis improving	[107]
*R. opacus* PD630	*tadA* disruption and overexpression	Gene role evaluation in LDs ontogeny. Effect on TAG accumulation	[108]
*R. opacus PD630*	*tadD* disruption and overexpression	Gene role evaluation in TAG biosynthesis. TAG accumulation improving from NADPH supply	[13]
*R. opacus* PD630	Disruption and overexpression of *fasI* gene	Gene role evaluation in TAG biosynthesis. TAG accumulation improving, under nitrogen rich-conditions	[109]
*R. opacus* PD630	Acyl-coenzyme A (CoA) synthetases deletion and overexpression of three lipases.	Free fatty acids (FFAs) production	[110]
*R. opacus* PD630	Acyl-CoA dehydrogenases deletion, overexpression of lipases, foldase, acyl-CoA synthetase and heterologous expression of an aldehyde/alcohol dehydrogenase and wax ester synthase	Fatty acid ethyl esters (FAEEs) production	[110]
*R. opacus* PD630	Acyl-CoA dehydrogenases and alkane-1 monooxygenase deletion, overexpression of lipases, foldase, acyl-CoA synthetase and heterologous expression of acyl-CoA reductase, acyl-ACP reductase, and aldehyde deformylating oxygenase	Long-chain hydrocarbons (LCHCs) production	[110]
*R. jostii* RHA1	Disruption and overexpression of *atf8* gene	Gene role evaluation in TAG biosynthesis	[16]
*R. jostii* RHA1	Disruption and overexpression of *nlpR* gene	Gene role evaluation in TAG biosynthesis. TAG accumulation improving, under nitrogen rich-conditions	[20]
*R. jostii* RHA1	Deletion and overexpression of *pap2* gene	Gene role evaluation in TAG biosynthesis. TAG accumulation improving from DAG supply	[111]
*R. jostii* RHA1	Overexpression of *ME-NADP^+^* gene (malic enzyme)	Gene role evaluation in TAG biosynthesis. TAG accumulation improving from NADPH supply	[14]
*R. jostii* RHA1	Overexpression of *ltp1* gene	Gene role evaluation in TAG biosynthesis. TAG and cellular biomass improving from fatty acids	[112]
*R. jostii* RHA1	*mlds* deletion	Gene role evaluation in LDs ontogeny. Effect on TAG accumulation	[113]

## Data Availability

Not applicable.
